# Mineral and phytate contents of some prepared popular Ghanaian foods

**DOI:** 10.1186/s40064-016-2202-9

**Published:** 2016-05-10

**Authors:** George Amponsah Annor, Kwaku Tano Debrah, Allen Essen

**Affiliations:** Department of Nutrition and Food Science, University of Ghana, P.O. Box LG 134, Legon-Accra, Ghana

**Keywords:** Mineral, Ghanaian, Dishes, Phytate, Popular, Iron, Zinc, Calcium

## Abstract

Prepared Ghanaian traditional foods, mostly consist of starchy staples such as yams (*Dioscorea* spp.), cassava (*Manihot esculenta*), millet (*Pennisetum glaucum*), maize (*Zea mays*) and rice (*Oryza sativa*) etc. These traditional foods are a main source of energy and macronutrients. Little or no information however exist on the mineral and phytate contents of prepared traditional Ghanaian foods. The mineral and phytate contents of twenty commonly eaten Ghanaian foods, prepared using popular recipes were analysed for their Fe, Cu, Zn, Mg, Mn, Ca, Na and K as well as phytate contents after foods were dried. Sodium was high in most of the foods, ranging from 557 mg/100 g for *Akple* with *okro* soup, to 193.7 for *Kooko* and bread. Boiled cowpeas with fried plantain was found to contain the highest amount of potassium (409.0 mg/100 g) followed by *konkonte* with groundnut soup (384.7 mg/100 g). Kooko with bread recorded the lowest potassium content of 131.72 mg/100 g. *Konkonte* with palm-nut soup and also with groundnut soup were among the foods found to contain high amounts of iron (14.1 mg/100 g and 13.2 mg/100 g respectively). All the foods were very good sources of minerals and will significantly contribute to the mineral intakes of consumers; however, their sodium contents were of concern.

## Background

Ghana is known throughout West Africa for the variety and quality of its traditional cuisine. Across the country, different ethnic groups or tribes prepare foods differently with varied ingredients. These differences largely reflect the types of crops produced in the various regions of the country (Salm and Falola 2002). Ghanaian traditional foods consist mainly of starchy staples such as yams, cassava, millet, maize and rice etc. These staples are usually served with spicy soups or stews and portions of meat or fish. Common spices used in these Ghanaian dishes are ginger, garlic, chillies and nutmeg etc. Wild mushrooms, garden eggs (similar to eggplant) are examples of vegetables used. Some examples of common traditional Ghanaian foods are *waakye* (cooked rice and cowpeas), *fufu* (cooked and pounded cassava and plantain or cooked and pounded yam and plantain, or cooked and pounded cocoyam), *banku* (cooked fermented maize dough and cassava dough), *kenkey* (cooked fermented maize dough wrapped in dried corn shooks or banana leaves), *kokonte* (cooked paste from fermented cassava flour) and *gari* (roasted cassava grits). Phytic acid is found in most cereal grains, legumes, nuts, oilseeds, tubers, pollen, spores, and organic soils. It acts as the primary phosphorus reserve accounting for up to 85 % of the total phosphorus in cereals and legumes (Tsao et al. 1997). Since phytates cannot be absorbed, and humans have a limited capacity for hydrolysing the phytate molecule, a negative effect of phytic acid on mineral bioavailability can be expected (Lönnerdal et al. 1989). Minerals represent about 0.2–0.3 % of the total intake of all nutrients in our diets and are necessary for the maintenance of normal cellular metabolism and tissue function (Sikorski 2006). Some attempts have been made to determine the mineral contents of some Ghanaian foods. A look through literature reveals little information on the mineral contents of prepared Ghanaian foods. The few studies available focused on ingredients rather than prepared foods. This study therefore aims at investigating the mineral of some prepared popular Ghanaian foods.

## Results and discussion

The minerals contents of the 20 popular Ghanaian foods, expressed on a dry weight basis in mg per 100 g edible portion are presented in Table 1. The phytate content, phytate:zinc, phytate:iron, calcium:phytate and (calcium × phytate):zinc molar ratios for the food samples are also presented in Table 2.

**Table 1:** Mineral (Fe, Cu, Zn, Ca, Mg, K, Mn and Na) contents of popular Ghanaian foods

Foods	Moisture (g/100 g)	Iron (mg)	Copper (µg)
Yam with *kontomire* stew	93.9 ± 0.2	6.6 ± 0.1	60 ± 10
Yam with garden egg stew	92.1 ± 0.2	3.6 ± 0.1	70 ± 20
Plantain with *kontomire* stew	93.3 ± 0.1	7.1 ± 0.1	ND
Plantain with garden egg stew	91.5 ± 0.2	4.1 ± 0.1	10 ± 10
*Fufu* with light soup	86.3 ± 0.1	8.1 ± 0.1	360 ± 20
*Fufu* with palm-nut soup	93.4 ± 0.1	6.3 ± 0.2	440 ± 20
*Fufu* with groundnut soup	87.1 ± 0.2	10.5 ± 0.2	500 ± 10
*Konkonte* with palm-nut soup	93.5 ± 0.2	14.1 ± 0.5	ND
*Konkonte* with groundnut soup	92.7 ± 0.3	13.2 ± 0.4	ND
*Akple* with okro soup	91.0 ± 0.1	5.3 ± 0.6	ND
*Kooko* with bread	91.4 ± 0.2	4.8 ± 0.2	ND
*Kenkey* with fried fish and pepper	94.3 ± 0.2	13.0 ± 0.3	ND
*Jollof* rice	95.1 ± 0.1	4.8 ± 0.0	ND
Plain rice and stew	95.0 ± 0.1	4.3 ± 0.0	ND
*Omo tuo* with palm-nut soup	91.4 ± 0.2	12.5 ± 0.3	ND
*Omo tuo* with groundnut soup	86.9 ± 0.1	11.1 ± 0.1	500 ± 10
*Waakye* with stew	95.1 ± 1.0	5.3 ± 0.1	1550 ± 310
*Hausa Kooko* with bread and akara	91.1 ± 0.1	15.5 ± 0.3	20 ± 2
*Tuo zaafi*	90.9 ± 0.2	7.7 ± 0.0	ND
Beans with fried plantain	94.5 ± 0.2	8.0 ± 0.2	90 ± 9

Foods	Zinc (mg)	Calcium (mg)	Magnesium (mg)
Yam with *kontomire* stew	2.5 ± 0.1	103.6 ± 0.8	43.3 ± 0.8
Yam with garden egg stew	2.2 ± 0.1	13.5 ± 0.1	41.2 ± 0.7
Plantain with *kontomire* stew	2.01 ± 0.1	116.1 ± 0.9	46.4 ± 0.3
Plantain with garden egg stew	1.7 ± 0.1	26.0 ± 0.2	44.2 ± 0.2
*Fufu* with light soup	3.8 ± 0.1	12.2 ± 0.2	42.7 ± 0.5
*Fufu* with palm-nut soup	2.2 ± 0.1	27.5 ± 0.3	45.3 ± 0.4
*Fufu* with groundnut soup	4.2 ± 0.1	10.6 ± 0.2	44.5 ± 0.3
*Konkonte* with palm-nut soup	3.3 ± 0.0	146.7 ± 0.5	44.6 ± 0.2
*Konkonte* with groundnut soup	3.1 ± 0.1	158.2 ± 05	47.1 ± 0.1
*Akple* with okro soup	3.0 ± 0.1	14.5 ± 0.1	45.7 ± 0.1
*Kooko* with bread	1.1 ± 0.0	4.9 ± 0.0	35.8 ± 0.3
Kenkey with fried fish and pepper	2.8 ± 0.1	100.0 ± 1.0	45.3 ± 0.4
*Jollof* rice	3.5 ± 0.0	9.6 ± 0.1	39.0 ± 0.5
Plain rice and stew	2.8 ± 0.1	59.8 ± 0.3	41.8 ± 0.6
*Omo tuo* with palm-nut soup	2.7 ± 0.1	48.7 ± 0.3	45.5 ± 0.5
*Omo tuo* with groundnut soup	4.7 ± 0.1	9.8 ± 0.2	38.5 ± 0.3
*Waakye* with stew	2.9 ± 0.1	24.7 ± 0.2	46.7 ± 0.2
*Hausa kooko* with bread and akara	2.3 ± 0.1	9.9 ± 0.1	42.2 ± 0.2
*Tuo zaafi*	4.0 ± 0.1	28.7 ± 0.2	46.8 ± 0.6
Beans with fried plantain	2.9 ± 0.0	21.3 ± 0.2	46.5 ± 0.1

Foods	Potassium (mg)	Manganese (mg)	Sodium (mg)
Yam with *kontomire* stew	374.0 ± 1.8	0.7 ± 0.1	443.3 ± 1.3
Yam with garden egg stew	367.7 ± 1.5	0.4 ± 0.0	423.0 ± 1.0
Plantain with *kontomire* stew	371.0 ± 1.8	0.8 ± 0.1	409.2 ± 1.5
Plantain with garden egg stew	364.7 ± 1.5	0.5 ± 0.0	388.8 ± 1.3
*Fufu* with light soup	379.8 ± 2.6	0.6 ± 0.1	360.8 ± 1.8
*Fufu* with palm-nut soup	351.8 ± 2.1	0.6 ± 0.0	303.8 ± 1.8
*Fufu* with groundnut soup	353.5 ± 1.8	0.7 ± 0.1	367.3 ± 1.8
*Konkonte* with palm-nut soup	340.3 ± 1.5	0.8 ± 0.1	353.3 ± 2.1
*Konkonte* with groundnut soup	384.7 ± 1.5	0.9 ± 0.1	409.7 ± 1.5
*Akple* with okro soup	330.0 ± 1.0	0.7 ± 0.1	556.7 ± 1.5
*Kooko* with bread	131.7 ± 1.5	0.5 ± 0.0	193.7 ± 1.5
*Kenkey* with fried fish andpepper	266.7 ± 1.5	0.7 ± 0.6	498.3 ± 1.5
*Jollof* rice	235.7 ± 1.5	0.8 ± 0.0	507.0 ± 1.0
Plain rice and stew	185.7 ± 1.5	0.8 ± 0.1	442.0 ± 1.0
*Omo tuo* with palm-nut soup	230.0 ± 2.0	1.0 ± 0.0	556.0 ± 1.0
*Omo tuo* with groundnut soup	205.0 ± 0.2	0.8 ± 0.5	511.2 ± 1.5
*Waakye* with stew	292.3 ± 2.1	1.1 ± 0.1	523.0 ± 1.0
Hausa kooko with bread and *akara*	166.7 ± 1.5	1.2 ± 0.1	236.0 ± 1.0
*Tuo zaafi*	376.0 ± 2.0	1.3 ± 0.2	492.0 ± 1.0
Beans with fried plantain	409.0 ± 1.0	1.1 ± 0.2	364.7 ± 1.5

Means ± standard deviations

*ND* not detected

**Table 2:** Concentration of phytate and phytate:zinc, phytate:iron, calcium:phytate and [Ca] [phytate]:[Zn] molar ratios of the popular Ghanaian foods

Dishes	Phytate (mg/100 g)	Phy:Zn	Phy:Fe	Ca:Phy	[Ca × Phy]:[Zn]
Yam with *kontomire* stew	15.72	0.6	0.2	107.9	0.02
Yam with garden egg stew	8.75	0.4	0.2	25.4	0.001
Plantain with *kontomire* stew	7.15	0.4	0.1	267.2	0.01
Plantain with garden egg stew	0.18	0.01	0.004	2377.8	0
*Fufu* with light soup	9.11	0.2	0.1	22	0.001
*Fufu* with palm nut soup	9.11	0.4	0.1	49.7	0.002
*Fufu* with groundnut soup	8.93	0.2	0.1	19.5	0
*Konkonte* with palm nut soup	0	0	0	0	0
*Konkonte* with groundnut and soup.	1.79	0.1	0.01	1455.4	0.002
*Akple* with okro soup	18.57	0.6	0.3	12.7	0.002
*Kooko* with bread	5.36	0.5	0.1	15.1	0.001
Kenkey with fried fish and pepper	20	0.7	0.1	82.3	0.02
*Jollof* rice	6.07	0.2	0.1	26	0
Plain rice and stew	10	0.4	0.2	98.5	0.01
*Omo tuo* with palm-nut soup	15.71	0.6	0.1	51	0.01
*Omo tuo* with groundnut soup	8.4	0.2	0.1	19.2	0
*Waakye* with stew	17.5	0.6	0.3	23.2	0.003
*Hausa kooko* with bread and *akara*	20	0.9	0.1	8.2	0.002
*Tuo zaafi*	12.5	0.3	0.1	37.9	0.002
Beans with fried plantain	12.14	0.4	0.1	28.8	0.002

The Phy:Zn, Phy:Fe, Ca:Phy and [Ca × Phy]:[Zn] values were calculated using the method of Wyatt and Triana-Tejas (1994)

### Iron

The iron content of foods analysed ranged from 3.6 to 15.5 mg/100 g, with the highest being recorded for *Hausa kooko* with bread and *akara,* and the lowest in yam with garden-egg stew. *Konkonte* with palm nut soup and also with groundnut soup were among the foods found to contain high amount of iron at 14.1 mg/100 g and 13.5 mg/100 g respectively. Plantain with garden-egg stew (4.1 mg/100 g), *kooko* with bread (4.8 mg/100 g) and plain rice and stew (4.3 mg/100 g) contained quite low amounts of iron compared to the rest of the dishes. The high iron contents for some of the dishes may be due to the relatively high amounts of animal proteins (meat and fish) in these dishes. For example: *konkonte* with palm-nut soup contained 90 g of tuna and 47 g of meat and had an iron content of 14.1 mg/100 g. There is evidence that phytic acid has a very marked inhibitory effect on the absorption of non-haem iron in man (Sandberg and Svanberg 1991), therefore, the high level of phytic acid (20 mg/100 g) detected in *hausa kooko* with bread and *akara* as well as *kenkey* with fried fish and pepper suggest that phytic acid may have an effect on the absorption of iron from these foods. The foods analysed had varied but high iron contents and consequently phytate:iron molar ratios were below 1.0 (Table 2). According to Hurell (2003), phytate begins to lose its inhibitory effect on iron absorption when phytate:iron molar ratios is less than 1.0. This study suggests that the absorption of intrinsic non-haem iron from these foods may not be significantly inhibited by phytate. Furthermore, the animal proteins (fish and meat) present in some of these dishes can counteract the inhibitory effect of phytic acid on iron absorption from the diet (Reddy et al. 1996).

### Copper

*Waakye* with stew (1550 µg/100 g) had the highest amount of copper followed by *fufu* with groundnut soup and *omo tuo* with groundnut soup, both recording about 500 µg/100 g of copper. The lowest amount of copper was observed in plantain with garden-egg stew (10 mg/100 g). *Hausa kooko* with bread and *akara*, yam and *kontomire* stew and yam with garden-egg stew were equally found to be poor sources of copper (20, 60 and 70 µg/100 g respectively). In general, the copper content of the foods analysed in this study was quite low with copper was not detected in nine out of the twenty foods.

### Zinc

*Omo tuo* with groundnut soup had a zinc content of 4.7 mg/100 g, followed by *fufu* with groundnut soup (4.2 mg/100 g) and *tuo zaafi* (4.0 mg/100 g). The lowest level of zinc was seen in *kooko* with bread (1.1 mg/100 g) and plantain with garden egg stew (1.7 mg/100 g) as shown in Table 1. Because phytate is a major inhibitor of zinc absorption, the phytate:zinc molar ratio is used to estimate the likely absorption of zinc from a mixed diet (Shils et al. 2006). The phytate:zinc molar ratio of all the dishes analysed in this study were low (Table 2) and hence, a sign of good bioavailability. About 4.7 mg/100 g of zinc was recorded for o*mo tuo* with groundnut soup.

### Calcium

The calcium content of the dishes ranged from a high of 158.0 mg/100 g in *konkonte* with groundnut soup to a low of 4.9 mg/100 g in *kooko* with bread. *Konkonte* with palm nut soup (146.7 mg/100 g), plantain and *kontomire* stew (116.1 mg/100 g), yam with *kontomire* stew (103.6 mg/100 g) and *kenkey* with fried fish and pepper sauce (100.0 mg/100 g) had high calcium contents. Even though calcium is the most abundant mineral in the body, it is present in significant amount in very limited number of foods (Dunne 1990). Wise (1983) suggested that the solubility of phytates and the proportion of zinc bound in a mineral complex in the intestines depend on the levels of calcium. In his model, phytate precipitation is not complete until dietary calcium:phytate molar ratios attain a value of approximately 6:1. Based on this observation, the high calcium:phytate molar ratios observed in these foods may lead to a reduction in the level of bioavailable zinc when consumed. The calcium:phytate molar ratios of all the dishes, except *konkonte* with palm nut soup, were greater than 6:1 and this, according to Wise (1983), is regarded as unfavourable for calcium absorption from these foods. Even though, the low calcium content of some of the foods is undesirable, it is of an advantage because, high calcium content may also jeopardize bioavailability of iron (Hallberg and Hulthein 2000) and zinc (Wise 1983). According to Ellis et al. (1987) and Davies and Warrington (1986), if the (calcium × phytate): zinc values are >0.50 mol/kg there will be interference with zinc availability which will lead to increased risk of deficiency. High calcium levels in foods can promote the phytate-induced decrease in zinc bioavailability when the calcium × phytate:zinc mill molar ratio exceeds 0.5 (Gibson 1994). Therefore, as values >0.5 were observed in these dishes, it would appear that the possible contribution of calcium in the diet in exacerbating the low bioavailability of zinc due to phytate is probably minimal.

### Magnesium

Magnesium was found to be high in *konkonte* with groundnut soup (47.1 mg/100 g) with the lowest occurring in *kooko* with bread (35.8 mg/100 g). High values were also recorded in foods such as *tuo zaafi* (46.8 mg/100 g), *waakye* with stew (46.7 mg/100 g) and also in plantain with *kontomire* (46.4 mg/100 g). *Konkonte* with groundnut soup appeared to contain the highest level of magnesium (47.1 mg/100 g). The high magnesium level observed in this particular dish could be due to the presence of groundnut which serves as the major ingredient in the preparation of the soup (Havel et al. 1989). The low levels of magnesium in some of the dishes (cereal based dishes) could also be attributed to the removal of the germ and outer layers of the cereal grains which accounts for more than 80 % loss in magnesium (Havel et al. 1989).

### Potassium

Cooked cowpeas (popularly called beans) with fried plantain was found to contain the highest amount of potassium (409.0 mg/100 g) followed by *konkonte* with groundnut soup (384.7 mg/100 g). *Tuo zaafi* (376.8 mg/100 g), yam with *kontomire* (374.0 mg/100 g), plantain with *kontomire* stew (371.0 mg/100 g) and *fufu* with light soup (3798 mg/100 g) all had an appreciable amount of potassium.

### Manganese

Most of the dishes had low levels of manganese except for 1.3 mg/100 g in *tuo zaafi*, 1.2 mg/100 g in *hausa kooko* with bread and 1.1 mg/100 g in beans with fried plantain. A lower value of 0.4 mg/100 g was observed in yam with garden egg stew and 0.5 mg/100 g in plantain with *kontomire* stew. The main staple food which forms the carbohydrate portion of *tuo zaafi* is millet (or corn flour in some cases) and this could possibly could account for the high manganese content, as whole grains and cereal products have been found to be the richest sources of manganese (Havel et al. 1989). Dishes which had their manganese levels between 1.0 and 1.2 mg/100 g such as *omo tuo* with palm nut soup (1.0 mg/100 g), *waakye* with stew (1.1 mg/100 g) and *hausa kooko* with bread and *akara* (1.2 mg/100 g) also have cereals forming the main portion of these foods.

### Sodium

Sodium was quite high in most of the dishes analysed. Values from 556.7 mg/100 g in *akple* with *okro* soup, 556.0 mg/100 g in *omo tuo* with palm nut soup and 507.0 mg/100 g in *jollof* rice were observed. The lowest amount of sodium, 193.7 mg/100 g, was recorded in *kooko* with bread. *Akple* with *okro* soup, *omo tuo* with palm nut soup and j*ollof* rice had the highest amount of sodium (556.7 mg/100 g), (556.0 mg/100 g) and (507.0 mg/100 g) respectively, which were also quite higher than the safe and adequate intake levels of 500 mg/day. The remaining dishes had lower amounts of sodium which can be described as desirable since sodium reduction in the diet is recommended as means of preventing hypertension and subsequent cardiovascular disease, stroke and renal failure (Sikorski 2006).

### Phytate

The level of phytate in the foods were high, ranging from about 20.0 mg/100 g in *kenkey* with fried fish and pepper sauce as well as *hausa kooko* with bread and *akara*. *Konkonte* with palm nut soup had no phytates. This could probably be due to the fact that *konkonte* is prepared from fermented cassava. Fermentation is known to reduce the phytates in foods. All the dishes showed marked variability in their level of phytate. Maize and millet goes through the process of soaking in water, milling and fermentation, before they are finally used in the preparation/cooking of *kenkey* and *hausa kooko* respectively. This might have accounted for the observed low level of phytate in these foods.

## Conclusions

This study has established that the popular prepared Ghanaian foods are very good sources of iron, copper, zinc, calcium, magnesium, potassium and manganese. Most of these foods are also likely to meet more than 100 % of the daily requirements (RDA) of these minerals. The sodium contents in these diets was however of a concern considering the implications on the consumption of high sodium consumption on heart health.

## Materials and methods

### Preparation of meals

The preparation methods used for the chosen recipes are those described by Manu (2006). Ingredients used for the preparation of the meals were purchased from three local markets; *Agbogbloshie*, *Madina* and *Kaneshie* markets in the Greater Accra region. The meals were prepared in two commercial kitchen facilities, both located at the University of Ghana. A summary of the ingredients and method of preparation of the various dishes is shown in Table 3.

**Table 3:** Description of standardized foods

Foodstuff	Food	Description
(a) Yam (*Dioscorea rotundata*)	Yam and *nkontomire* stew	Boiled yam with stew prepared from boiled cocoyam leaves, tomatoes, onion and palm oil with fried/smoked fish
	Yam and garden egg stew	Boiled yam with stew prepared from boiled and ground garden eggs, tomatoes, onion, pepper and palm oil with fried and smoked fish
(b) Plantain (*Musa sapientum*)	Plantain and *nkontomire* stew	Boiled plantain with stew prepared from cocoyam leaves, tomatoes, onion and palm oil with fried/smoked fish
	Plantain and garden egg stew	Boiled plantain with stew prepared from boiled and ground garden eggs, tomatoes, onion, pepper and palm oil with fried and smoked fish
(c) Cassava (*Manihot esculenta*)	*Fufu* with palm nut soup	Boiled cassava and plantain was pounded to a smooth paste in a wooden mortar. Soup contained tomatoes, onions, pepper, garden eggs, salt, water and fish/meat
	*Fufu* and peanut soup	Boiled cassava and plantain was pounded to a smooth paste. Soup was prepared from peanut paste, tomatoes, pepper, garden eggs, salt, water and fish/meat
	*Konkonte* and palm nut soup	Fermented cassava flour cooked to a paste with water. Soup contained tomatoes, pepper, onions, salt, water and fish/meat
	*Konkonte* and peanut soup	Fermented cassava flour cooked to a paste with water. The soup was prepared from peanut paste, tomatoes, pepper, garden eggs, salt, water and fish/meat
(d) Maize (*Zea mays*)	*Akple* and *okro* soup	Cooked non-fermented corn dough and cassava dough. Soup contained *okro*, tomatoes, pepper, garden eggs, onions, salt and smoked/grilled fish or meat
	*Kooko* with bread (fermented corn meal porridge)	Porridge prepared from fermented corn dough and served with bread
	*Kenkey* with fried fish and pepper.	Fermented corn dough boiled and stirred into a smooth paste. Paste was moulded into balls, wrapped in clean corn shooks and steamed for 2–3 h. It was served with fried fish, sliced onions and pepper sauce
(e) Rice (*Oryza sativa*)	*Jollof* rice and stew	Rice cooked with tomatoes, pepper, salt, onions, meat, lard, thyme, water and meat
	Plain rice and stew	Boiled rice served with stew prepared from tomatoes, onions, pepper, salt, cooking oil and fish/meat
	*Omo tuo* with palm nut soup	Cooked rice made into balls and served with palm nut soup (as described above)
	*Omo tuo* with groundnut soup	Cooked rice made into balls and served with groundnut soup
	*Waakye* with stew	Rice cooked together with beans and served with macaroni, gari and stew prepared with tomatoes, onions, pepper, salt, cooking oil and fish/meat
(f) Millet (*Pennisetum typhoides*)	*Hausa kooko* with bread and *akara*	Ground millet and about one-third pepper cooked with water to form porridge and served with sugar, bread and *akara*
	*Tuo Zaafi*	Boiled and thickened porridge ball prepared from millet and served with soup containing tomatoes, pepper, onions, *okro*, *kontomire* leaves and fish/meat
(g) Cowpea (*Vigna ungiculata*)	Beans and fried ripe plantain (*red*–*red*)	Cooked beans served with palm oil, gari and fried ripe plantain

### Sample collection and preparation

Each of the meals were prepared separately in the different commercial kitchens. After the preparation of the meals, each meal was cooled to room temperature and the whole meal homogenized using a waring blender. The homogenized meals were dried in an air-oven at 50 °C overnight and ground into flour using a mortar and pestle. The dried meals were packed in moisture resistant polyethylene bags and kept at approximately 4 °C for further analysis. The meals from the different commercial kitchens were analysed separately and means of the different analysis found. The preparations were done twice in the separate kitchens.

## Methods

### Moisture determination

The moisture contents of the samples were determined using the air-oven method described by Osborne and Voogt (1978). Moisture dishes were cleaned, dried in oven and labelled for identification. Dishes together with the lid were weighed after cooling in a desiccator to determine the exact weight. In the method, approximately 2 g of the sample was weighed into each moisture dish. Samples were then dried at 105 °C overnight in the air oven leaving the covers slightly opened. Then content of the moisture dishes was then cooled in a desiccator and weighed using an electronic weighing balance (AFA 210 LC, Adam Equipment). The percentage moisture determined was then used to calculate the percentage total solids content of the samples.

### Wet digestion of sample

The first step involved in the determination of the inorganic materials was through the procedure of wet ashing. The AOAC 1990 method was employed. The glass wares were acid-washed overnight in 2 M HCL rinsed with distilled water and the oven dried 1 g of sample was weighed (Weighing balance, AFA 210 LC, Adam Equipment) into a dry acid-washed 250 ml beaker. Twenty-five ml (25 ml) of concentrated nitric acid was added and the beaker covered with a watch glass. The sample was digested with great care on hot plot (Hot plate, Sand Juniper and Co. Harlow, Essex, England) in a fume chamber until the solution was pale yellow. The solution was cooled and 1 ml Perchloric acid (70 % *HCLO*_4_) added. The digestion was continued until the solution was colourless or nearly so (the evacuation of dense fumes indicates the removal of nitric acid). When the digestion was completed, the solution was cooled slightly and 30 ml of deionised water added. The mixture was brought to boiling for 10 min and filtered while hot, into a 100 ml volumetric flask using a Whatman No. 4 filter paper. The solution was then made to the mark with deionised water. Flasks were stoppered and stored in a cold room at 10 °C awaiting mineral analysis.

### Determination of Fe, Cu, Zn, Mg, Mn and Ca

One ml of the digest was used to determine the Fe, Cu, Zn, Mg, Mn and Ca contents of the samples using the Perking Elmer Precisely A Analyst 400 Atomic Absorbance Spectrophotometer with an acetylene flame. The AAS was fitted with Zn and Fe EDL lamps and Mg, Mn, Cu and Ca CHCL lamps set at wavelengths of 213.86, 248.33, 285.21, 279.5, 324.75, 422.67 λ respectively.

### Determination of Na and K

Two (2) ml of the digest was used in the determination of sodium and potassium using the flame photometric method. The photometer (Jenway, United Kingdom) model PF P7 with methane gas was used for this analysis.

### Phytate determination

Phytate determination in the food samples followed the method of Wheeler and Ferrel (1971). Ten (10) g of a finely ground sample (40 screen) estimated to contain 5–50 g P-phytate was weighed into a 125 ml Erlenmeyer flask. Phytate was extracted with 50 ml 3 % solution of trichloroacetic acid for 30 min with constant agitation. The resulting suspension was centrifuged (Denley BS400 Centrifuge, Payne, England) at 160 revolutions per minute for 30 min and a 10 ml aliquot of the supernatant liquid was transferred into a conical centrifuge tube. 4 ml of *FeCl*_3_ solution prepared to contain 2 mg *Fe*^3+^ was added, blowing through the pipette rapidly. The centrifuge tube containing the sample was heated for 45 min in a water bath (Nickel Electronics Ltd, England) at 90–100 °C. (When the supernatant was not clear after 30 min, 2 drops of the solution of sodium sulphate at 3 % in trichoroacetic acid (TCA) was added and heated for 10 more minutes). The solution was centrifuged for 15 min at 160 revolutions per minutes and the supernatant liquid was carefully poured off. The remaining precipitate was washed twice with 20 ml TCA at 3 % solution, dispersing it well and heating for 10 min in boiling water and centrifuging for 15 min at 160 revolutions per minute. The wash was finally repeated once more with distilled water. The resulting precipitate was dispersed in a little distilled water followed by the addition 3 ml of 1.5 N NaOH solution and stirring. The volume of the solution was brought to approximately 30 ml with distilled water and heated in boiling water for 30 min. The solution was filtered whilst hot with a Whatman No. 4 filter paper. The precipitate was washed with 60 ml of hot distilled water and the filtrate was discarded. The precipitate left in the paper was dissolved with 40 ml of 3.2 N solution of *HNO*_3_ transferring it to a 100 ml volumetric flask. The paper was washed several times with distilled water, collecting it in the flask. The sample was then cooled to room temperature and calibrated with distilled water. Five (5) ml of this solution was transferred into a 100 ml volumetric flask and diluted to approximately 70 ml with distilled water. 20 ml of 1.5 N KSCN was then added and the solution was made up to the 100 ml mark with distilled water. Absorbance of the solution was read (approximately within 1 min) in a spectrophotometer (Spectrophotometer UV-120-02, Shimadzu Cooperation, Japan) at 480 nm. All readings were corrected by the reading of a blank carried out alongside each set of samples in order to eliminate the effect of any colour produced by the reagents. A standard curve colorimetric reading versus concentration of *Fe*^3+^ using portions of standard iron solutions (2, 4, 6, 8, 10 and 12 ml) subjected to reaction with potassium thiocynate was drawn, from which the concentrations of iron was obtained. The phytate content was then calculated from the iron concentration by assuming a constant Fe:P molecular ratio of 4:6 in the precipitate.

### Laboratory precautions

To minimize the risk of adventitious contamination, all glassware used for the analytical methods for the analytical methods was acid-washed, and sterile disposable powder-free plastic gloves were worn when handling the foodstuffs during the sampling and analysis stages.
